# The ethical canary: narrow reflective equilibrium as a source of moral justification in healthcare priority-setting

**DOI:** 10.1136/jme-2023-109467

**Published:** 2024-02-19

**Authors:** Victoria Charlton, Michael J DiStefano

**Affiliations:** 1 Global Health and Social Medicine, King's College London School of Social Science and Public Policy, London, UK; 2 Department of Clinical Pharmacy, University of Colorado, Aurora, Colorado, USA

**Keywords:** Public Policy, Ethics- Medical, Morals, Philosophy, Resource Allocation

## Abstract

Healthcare priority-setting institutions have good reason to want to demonstrate that their decisions are morally justified—and those who contribute to and use the health service have good reason to hope for the same. However, finding a moral basis on which to evaluate healthcare priority-setting is difficult. Substantive approaches are vulnerable to reasonable disagreement about the appropriate grounds for allocating resources, while procedural approaches may be indeterminate and insufficient to ensure a just distribution. In this paper, we set out a complementary, coherence-based approach to the evaluation of healthcare priority-setting. Drawing on Rawls, we argue that an institutional priority-setter’s claim to moral justification can be assessed, in part, based on the extent to which its many normative commitments are mutually supportive and free from dissonance; that is, on the ability to establish narrow reflective equilibrium across the normative content of a priority-setter’s policy and practice. While we do not suggest that the establishment of such equilibrium is sufficient for moral justification, we argue that failure to do so might—like the proverbial canary in the coalmine—act as a generalised warning that something is awry. We offer a theoretical argument in support of this view and briefly outline a practical method for systematically examining coherence across priority-setting policy and practice.

## Introduction

 Institutions with a mandate to act as healthcare priority-setters undertake a difficult and unenviable task. Whether made on behalf of a public provider or private insurer, decisions about what healthcare interventions should be made available and to whom are often complex and contentious, with huge implications for those directly affected. Resource allocation decisions create both ‘winners’ and ‘losers’ from across society and often hinge on value judgements about which there is little agreement. Attempting to ensure that such decisions are accepted as legitimate is a challenge that all institutional priority-setters must face.

Such legitimacy might be derived in part through the legal and political status of the priority-setter and the authority of the political system that they operate within. However, ethics also plays an important role in securing legitimacy for priority-setting decisions. Even in countries where political legitimacy is high, resource allocation decisions can encounter strong societal resistance if they are perceived to be unjust.^1 2^ Such decisions may result in the decision-maker’s moral authority coming under question, to the detriment of legitimacy and its subsequent ability to make decisions that will be accepted without challenge.^3^ It is, therefore, prudent for a priority-setter to seek to justify its decisions on moral as well as legal and political grounds.

Priority-setters also plausibly have moral obligations that are independent of legitimacy, particularly when tasked with upholding population health. Health is central to well-being, the pursuit or protection of which holds primary moral importance in many ethical theories.^4^,^6^ Health is also critically important for ensuring fair equality of opportunity, providing a prima facie reason for priority-setters to avoid acting in ways that are to its detriment.^7^ Allocating resources based on arbitrary or unjustified reasons may breach a priority-setter’s institutional duty to act for the public good and, in some cases, would contravene morally grounded standards of public life for those working on a priority-setter’s behalf.^8^,^10^

Finding a normative basis from which to morally defend priority-setting decisions is, however, difficult. Individuals have different views on how resources should be allocated and any attempt to justify decisions with reference to substantive principles is, therefore, open to challenge by those who consider different principles to be more appropriate.^11^ In the absence of any single agreed theory of justice, such instances of reasonable disagreement cannot be reliably adjudicated, weakening claims to moral legitimacy. Many priority-setters have, therefore, opted to focus on procedure, reasoning that while individuals may differ in what they perceive to be just grounds for decision-making, we can all agree on what constitutes a just process. But it is questionable whether a just process by itself is sufficient to ensure a just outcome and experience has shown that even procedurally based approaches are liable to raise substantive moral questions.^12^,^14^ Thus, despite such efforts, evidence suggests that priority-setters are ‘falling short’ when it comes to demonstrating the legitimacy of their decisions.^15^

This paper builds on these arguments to offer a complementary basis for considering whether an institutional priority-setter is acting in ways that can be morally justified. It does so by suggesting that one characteristic of a morally just approach is that it is coherent: that is, that a priority-setter’s policy (as constituted by its formal processes and methods of appraisal) and practice (as constituted by the judgements made over the course of many individual cases) exist in a state of equilibrium.

We do not suggest that this type of equilibrium is sufficient for moral justification—we accept in principle that a priority-setter can act in ways both coherent and immoral. We also accept that the dynamic nature of any equilibrium will likely give rise to instances of transitory dissonance, as novel scenarios necessitate reflection on—and modification to—previously accepted policy and/or practice. But we argue that any claim for moral justification is undermined if substantial dissonance can be shown to persist within a priority-setter’s moral system. We, therefore, propose that the empirical examination of coherence is a useful approach to moral evaluation and that, like the proverbial canary in the coalmine, the inability to establish equilibrium might act as a generalised warning that something is awry .

The article proceeds as follows. In section 2, we expand on the problems associated with grounding moral claims entirely on substantive principles or on a particular substantive conception of justice. In section 3, we consider why procedural approaches might also by themselves be insufficient for moral justification. In sections 4 and 5, we introduce coherence as a complementary source of justification and explain why, in the context of healthcare priority-setting, the inability to establish narrow reflective equilibrium (NRE) might undermine a priority-setter’s claim to moral justification. In section 6, we outline a method for systematically examining coherence and briefly demonstrate how it might be used to explore the extent to which NRE can be established across priority-setting policy and practice. Finally, in section 7, we sum up our argument and conclude.

## The problem with substantive principles

Any decision, by definition, has content, and if a decision is to be non-arbitrary then that content must reflect substantive reasons. One approach to moral evaluation is to consider whether these reasons are morally justified. According to this rationale, if a priority-setter has made its decision for ‘good’ reasons, then it follows that the decision itself is ‘good’.

But what constitutes a good reason? One approach is to ground claims on substantive principles of justice.^16^ One might, for example, employ the utilitarian principle of health maximisation to argue that allocation decisions should be based on whether an intervention’s use will increase or decrease overall population health, once opportunity cost has been taken into account.^1^ Or one might take the prioritarian view that preference should be given to the worse-off in society, even if this is somewhat inefficient.^16^ Different principles generate different conclusions and, in the absence of an agreed theory of justice, there is no way to independently adjudicate between them: the utilitarian would simply argue that the prioritarian is mistaken in their moral beliefs and vice versa. Even under pluralist theories that incorporate a range of different principles, the question of how these should be selected, specified and balanced remains thorny. Thus, any priority-setter who seeks to justify their decisions on purely substantive grounds will likely find those grounds rejected by a proportion of society, undermining their legitimacy at least in the eyes of those who take an alternative view of what justice requires.

An alternative approach is to explicitly ground substantive principles for priority-setting on public views, for example, by conducting studies exploring societal values.^17^,^22^ Doing so might enhance a priority-setter’s perceived legitimacy and make its decisions less vulnerable to challenge. But the mere fact that a view is widely held does not guarantee that it is ethically permissible—a racist society may support a racially based allocation, but this would not make it morally justified. Moreover, recourse to public opinion offers no remedy for matters on which there is no public consensus. Reasonable disagreement has been demonstrated across a range of moral questions relevant to healthcare priority-setting and is the inevitable product of ethical deliberation in any free-thinking pluralist society.^22^,^27^ The problems associated with basing moral evaluation on individual substantive principles, therefore, remain unresolved.

## The limits of procedure

Given these challenges, many priority-setters have chosen to base their claim for moral legitimacy on use of a just procedure. The most widely adopted is the ‘accountability for reasonableness’ (AfR) framework developed by Daniels and Sabin, which has been implemented by several national and regional priority-setters since the late 1990s.^27 28^

Despite the popularity of procedural approaches, their ability to provide moral justification may be limited for a variety of theoretical and practical reasons. Procedural principles—like substantive principles—are subject to reasonable disagreement, leading to debate about the appropriate requirements of a fair procedure and the potential for legitimacy to be undermined if procedures are perceived by some to be unfair.^1214 26 29^,^32^ In the absence of detailed guidance about how such frameworks should be implemented, there can also be divergence in how procedural principles are specified in different contexts.^13 31 32^ Moreover, procedural approaches do not avoid the problems posed by substantive moral questions. In the case of AfR, such questions primarily concern what constitutes a ‘relevant’ (and therefore, according to AfR, an acceptable) reason for decision-making, with critics highlighting the indeterminacy of this requirement^12 30 33^ and the difficulties encountered when attempting to implement it in practice.^13^ Critics have also challenged AfR’s disregard for certain types of reason, such as those that are faith based, on the grounds that this itself reflects a substantive view that cannot be independently justified.^29^

Other less widely adopted procedural approaches might address some of these deficiencies. But it is not clear that any intended conception of pure procedural justice—AfR or otherwise—is applicable to healthcare priority-setting. In the paradigm case, the outcome of a coin toss could be considered to be just purely on the basis of the procedure followed, regardless of the substantive outcome—morally speaking, a head is no better than a tail.^34^ But healthcare priority-setting is not like coin tossing; some outcomes are morally better than others and unless priorities are to be determined by lottery, substantive reasons must be invoked in reaching a decision. A priority-setter’s use of a demonstrably fair procedure might therefore be effective in enhancing the legitimacy of its decisions. But this does not in itself provide strong grounds for claiming that a decision is morally justified.^14 30 33^

## Coherence and moral justification

A complementary approach lies in the notion of coherence: the idea that what vindicates us in considering certain beliefs to be morally justified is not their individual substance or the procedure used to reach them, but the relations of mutual support that exist between them.^35^ In the context of healthcare priority-setting, this implies that a priority-setter’s actions can be morally evaluated in part based on the extent to which its moral system as a whole is coherent.

A particularly influential conception of coherence is that proposed by Rawls as part of his theory of justice. According to Rawls, ‘a conception of justice cannot be deduced from self-evident premises or conditions on principles; instead, its justification is a matter of the mutual support of many considerations, of everything fitting together into one coherent view’.^36^ This ‘fitting together’ is achieved through the method of reflective equilibrium: a process in which ‘we start with our existing ethical beliefs about cases and principles, weed out those that are thought to be unreliable, and then adjust the remaining set in order to make it as coherent as possible’.^37^

The method of reflective equilibrium can be applied across differing realms of moral belief. In NRE, the aim is to achieve coherence across a set of considered moral judgements (a) and the general principles that explain them (b). While such an approach can be usefully applied to identify and resolve inconsistencies in a set of moral views, it has been argued that it does not provide any independent means of defending the credibility of these views, and therefore, has relatively limited justificatory power. Conversely, in a wide reflective equilibrium (WRE), relations of mutual support expand to encompass relevant background theories (c), providing further support for the conclusions generated. Thus, in WRE:

We do not simply settle for the best fit of principles with judgments […] which would give us only a narrow equilibrium. Instead, we advance philosophical arguments intended to bring out the relative strengths and weaknesses of alternative sets of principles (or competing moral conceptions). These arguments can be construed as inferences from some set of relevant background theories… In this way [one] arrives at an equilibrium point that consists of the ordered triple (a), (b), (c).^38^

It was Rawls’ view that only WRE, and not NRE, could be used to justify moral theory. However, he acknowledged that ‘it is doubtful whether one can ever reach this state’ of coherence in practice.^16^

## The canary in the coalmine

Despite Rawls’ misgivings about whether NRE is sufficient to justify a set of moral beliefs, others have argued that ‘moral justification is a matter of degree’^21^ and that ‘in the vast majority of cases […] it will probably turn out that NRE, for all its limitations, will be a tolerably helpful methodological tool’^39^ in conducting practical ethics.^40^ Given the many background theories that could be considered relevant to matters of healthcare priority-setting—theories of distributive and procedural justice, conceptions of health, ideas about moral personhood, principles of medical ethics and so on—an institutional priority-setter could not reasonably be expected to justify its approach on the grounds of having achieved WRE. But all that is required for a priority-setter to achieve the lesser goal of NRE is for it to ensure that its moral system comprises a set of mutually supportive normative commitments and does not contain persistent sources of dissonance.^41^^2^ Given the complexity of priority-setting methods and the large numbers of cases considered by institutional priority-setters, achieving even this narrow form of equilibrium would doubtless require significant effort and conscientious reflection. But this goal nevertheless appears to be eminently achievable and—employed alongside substantive and procedural considerations—would provide some confidence that a priority-setter’s actions could be morally justified. Conversely, the inability to achieve equilibrium across this relatively narrow realm might be considered akin to our ailing canary, signalling to those who observe it that there is likely a problem in the coalmine.

As has already been acknowledged, we do not suggest that achieving this relatively limited equilibrium is sufficient to conclusively demonstrate that a priority-setter’s approach is morally justified. However, on the view that moral justification is a matter of degree, achieving NRE is nevertheless a significant achievement indicative of moral reliability. Other factors may enable us to further increase confidence in our conclusions. Rawls derived much of the moral authority associated with achieving equilibrium from the fact that a coherent system has at its core considered moral judgements. Such judgements are more than simply hunches; though intuitive, they are reliable and stable conclusions that have arisen from the careful inquiry of ‘competent moral judges’.^42^ We might, therefore, be more confident in concluding that a priority-setter’s coherent approach is morally justified if it has been shaped by the collective views of a group whose members each display the qualities associated with a competent moral judge: qualities such as intelligence, empathy and impartiality.^42 43^^3^ Conversely, our confidence in this conclusion might be diminished if those who contribute to collective decision-making appear unpractised or unskilled in moral reflection, or if they have a vested interest in the outcome of the priority-setting exercise.

The confidence of our conclusions—and the ‘width’ of our equilibrium—might also be enhanced when a coherent moral system has been shaped by people with a broad range of experiences and beliefs, who are likely to draw on different background theories in making their considered moral judgements. For this reason, the equilibrium that emerges from a collectively developed approach informed by the independent conclusions of multidisciplinary decision-making committees might be considered wider and more morally reliable than one that arises through the work of a single institutional decision-maker.^4^ Similarly, all else being equal, an equilibrium that reflects the fair consideration of societal views—as evidenced, for example, through public dialogue or the deliberations of a Citizens Jury—might be considered to carry more justificatory force than one that has not involved external participation and conflicts with public opinion.^21^

If it is accepted that part of the justificatory power of coherence stems from the special status of considered moral judgements—that is, judgements that are reliable due to their ‘inherent plausibility, stability and low likelihood of being motivated by biases’^37^—then a potential criticism is that this introduces a substantive component to an approach badged as avoiding such complications. However, we would argue that the need for judgements to be morally reliable imposes a relatively loose constraint and does not, to any meaningful extent, turn our approach into a substantive one. The need for reliability may prohibit an allocation that is morally implausible, such as one based on race. But it is ambivalent about what role should be played in priority-setting by disease severity or patient age, for example, and does not seek to settle such matters of reasonable disagreement. Unlike other substantive approaches, therefore, our coherence-based approach does not independently ground justificatory claims on the substantive content of individual principles and allows that a wide variety of moral positions might be equally justified if they are similarly coherent and do not rest on claims that are morally implausible. Similarly, it avoids some of the limitations of procedural approaches, which base moral conclusions on the way that decisions are reached, rather than taking account of both procedural and substantive commitments in evaluating the coherence of a priority-setter’s moral system as a whole. As such, it provides a complementary means of morally evaluating healthcare priority-setting.

## A method for empirically examining coherence

As suggested above, the novelty of our contribution does not lie in the suggestion that coherence might be used as grounds for moral evaluation: normative claims based on coherence are well established in theory and commonly employed in empirical bioethics.^44^ Nor are we the first to see a role for reflective equilibrium in healthcare priority-setting: WRE has been used as a framework both for structuring priority-setting deliberations and for evaluating individual decisions.^45^,^50^ Very recently, coherence-based arguments have been employed in emphasising and characterising the role that empirical studies might play in justifying priority-setting principles.^21^ However, we believe that we are the first to suggest that coherence might be used as grounds for drawing evidence-based conclusions about the extent to which a priority-setter’s overall approach—incorporating both policy (as constituted by its formal processes and methods of appraisal) and practice (as constituted by the judgements made over the course of many individual cases)—is morally justified and to specifically propose that NRE be used as a tool in systematically examining coherence in this context.

Conducting such an examination is, however, a daunting task. Healthcare priority-setters generally conduct their work according to detailed processes and highly technical methods that incorporate many explicit and implicit normative commitments and are described in documents spanning hundreds of pages. A priority-setter’s body of decisions may consist of dozens of individual cases, each embedding many case-based judgements informed both by normative concerns and the analysis and interpretation of complex evidence, similarly described across hundreds of pages of documentation. Sources of normative dissonance may exist both within each of these realms (eg, in the form of conflicting aspects of policy or through inconsistent judgements made within and across cases) and between them (eg, through a failure to observe policy in practice or to update policy to reflect practised norms). To systematically examine coherence across this morass of empirical normative content, an organisational scheme is required.

**Figure 1:**
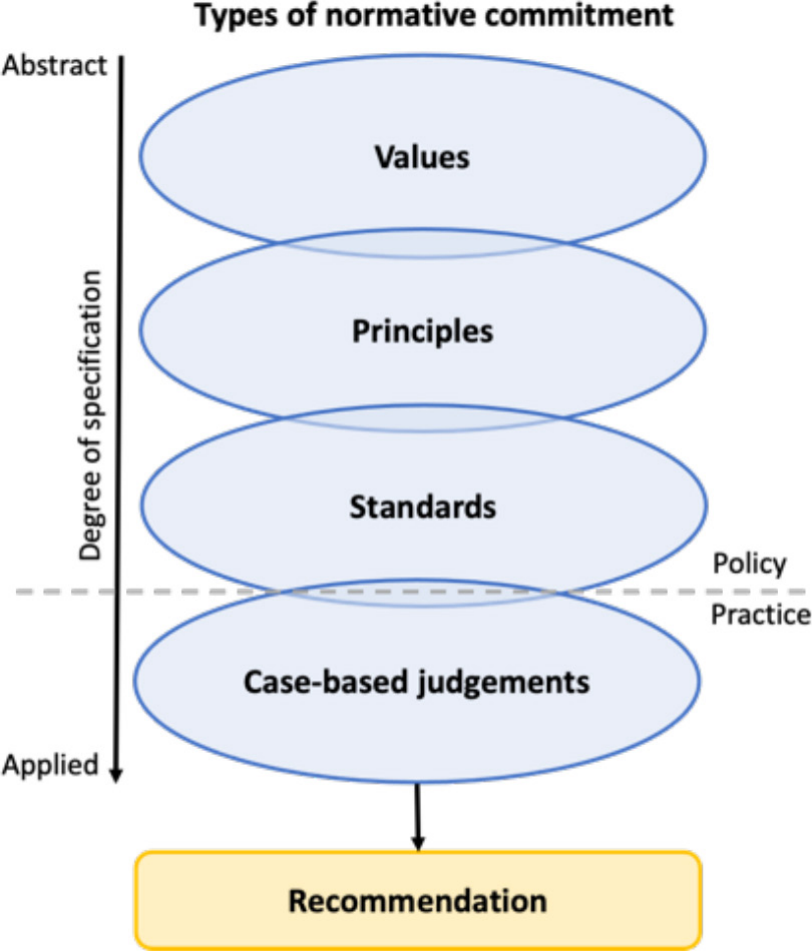
A framework for the articulation of normative reasoning in health technology assessment.

One such scheme, recently developed as a tool for articulating normative reasoning in healthcare priority-setting, classifies normative content according to four types, based on degree of specification (see figure 1).^51^ Values, the least specified normative type, describe abstract ends such as justice, liberty, dignity and happiness that a priority-setter believes are worth pursuing because they are ‘good’ or ‘right’. Principles are more specified action-guiding claims that serve as a pledge to act in a certain way or as the basis for part of a chain of reasoning. Standards are specific ways of doing things that are accepted by authority, precedent, custom or general consent. And case-based judgements are considered conclusions reached through the evaluation of information relevant to a specific context or case. Together, these four types of normative commitment give rise to a chain of reasoning from which is derived a priority-setting decision.^51^

This scheme allows coherence to be systematically evaluated by facilitating the classification of normative content, allowing the degree of alignment within and between each class to be assessed. Say, for example, that a priority-setter has promised to act in accordance with justice.^5^ In practice, the value of justice must be specified if it is to act as a guide to action, and this specification can take many different forms. Our hypothetical priority-setter specifies justice through two substantive principles: a principle of fairness, which stipulates that similar cases should be treated similarly, and a principle of efficiency, which requires it to recommend new interventions only if their adoption is likely to lead to a net increase in population health. These principles are operationalised through many detailed standards describing, for example, what procedures will be followed in reaching a decision, what characteristics of a case should be taken into account, how an intervention’s contribution to population health will be assessed and on what basis it will be deemed cost-effective. Ultimately, this policy—which can be shown to be coherent—is applied to an individual case, which generates a collection of further judgements that contribute to a decision about whether or not the intervention should be adopted.

To continue our simplified example, let us imagine that a well-resourced private US insurer is applying this policy in deciding whether to expand its coverage to a new cancer drug. Based on the available evidence, the drug is judged to be effective in extending patient survival and, at the given price, is found to meet the insurer’s formal standard for cost-effectiveness. The insurer has also recommended similar interventions in the past. In recommending this drug, therefore, the insurer would maintain equilibrium between the standards, principles and values that make up its existing (coherent) policy, and the judgements made in practice, both in this and previous cases. Let us say, however, that the drug does not meet the usual standard for cost-effectiveness, and the insurer decides to cover it anyway, despite previously rejecting similar cases. This judgement is not supported by either the insurer’s principle of efficiency or its principle of fairness, undermining its commitment to justice (as currently specified) and disrupting the equilibrium that previously existed between policy and practice. To reconstitute equilibrium and prevent legitimacy from being eroded through loss of moral authority, the insurer must amend one or more of the relevant principles and standards. If no morally reliable amendment can be made, it must be willing to reconsider its reasoning and, if necessary, modify this decision.^6^

Through such a process of iterative reflection and adjustment, a priority-setter can ensure that coherence is maintained across the many values, principles, standards and case-based judgements that make up its moral system, while also eliminating ambiguity and ensuring full transparency across its chain of reasoning.^51^ By empirically examining the relationships between these different types of normative commitment, those interested in evaluating this system can draw evidence-based conclusions about the extent to which it is morally justified and can make practical recommendations for how coherence might be enhanced. Such an approach might, therefore, be employed to improve the moral quality of decision-making while also supporting public acceptance of priority-setting decisions.

## Conclusion

This paper proposes that, in attempting to morally evaluate healthcare priority-setting, there is considerable merit in systematically assessing the coherence of a priority-setter’s moral system, thereby avoiding the limitations associated with substantive and procedural approaches to grounding moral claims. This is not to suggest that establishing NRE is sufficient for moral justification or that WRE does not hold greater justificatory force. But we argue that such coherence is a necessary and achievable condition of ethical decision-making and that, like our proverbial canary, the inability to establish NRE should be taken as a cause for concern.

Given the intense political and commercial pressures that institutional priority-setters often operate under, a worry when equilibrium cannot be achieved is that those acting in a decision-making capacity may not be exhibiting the qualities of competent moral judges and may be allowing ethically unreliable considerations—or vested interests—to impact on their moral thinking. While resource allocation is challenging for reasons that are social and political as well as ethical, we do not believe that this complexity absolves healthcare priority-setters from acting in ways that can be morally defended. We hope that the approach set out here provides a complementary means of ensuring that those tasked with making decisions on society’s behalf can be held properly and fully to account.

## Data Availability

No data are available.

## Footnotes

The opportunity cost associated with an intervention’s adoption refers to the benefits foregone when a choice is made to employ resources on adopting that intervention rather than for some other purpose within the health system.

Van Thiel and Van Delden use the following example, taken from Bonjour, to demonstrate what constitutes mutual support between beliefs: Proposition A: this chair is brown; electrons are negatively charged; today is Thursday. Proposition B: all ravens are black; this bird is a raven; this bird is black. Neither of these two propositions are logically inconsistent. But in proposition A consistency results from the fact that its components are almost entirely unrelated to each other – —although not inconsistent, they contain no relations of mutual support. This is not the case in proposition B, in which there are strong positive inferential relations between the premises. Thus, while proposition A merely demonstrates consistency, proposition B demonstrates coherence. Dissonance occurs where there are negative inferential relations between premises.

There is some intersection between Rawls’ notion of the competent moral judge and virtue ethics, in that competence is determined ‘“by the possession of certain characteristics, some of which may be said to be capacities and achievements (intelligence and knowledge), while others may be said to be virtues’ – —that is, ‘habits of thought and mind’ that ‘foster the conditions necessary for successful inquiry’. However, in Rawls’ view it is not the virtue of the individual thinker that contributes to moral justification, but the rational procedure through which the considered judgements of (often several) competent moral judges may inform the development of principles for deciding moral questions. Rawls refers to intelligence, empathy and impartiality as essential qualities of the competent moral judge. But others, such as Refolo *et al*, have posed other characteristics, such as integrity, courage and prudence, as core qualities of those directly involved in healthcare priority-setting.

Such a decision-maker might feasibly be an individual; for example, a senior organisational leader tasked with establishing a highly specified formal approach or even with making individual priority-setting decisions. However, in the institutional context, might also be made by a small but homogenous group; for example, an internal decision-making committee made up of senior figures.

The following example of how the framework might be applied in practice is brief and highly simplified. A more extended hypothetical example is included in the source paper.

An analogy might reasonably be drawn between this process and the establishment of legal precedent through the evolution of case law, particularly when normative precedents for healthcare priority-setting are established through the hearing of internal appeals or through formal legal proceedings such as judicial review.
